# COVID-19 and healthcare system in China: challenges and progression for a sustainable future

**DOI:** 10.1186/s12992-021-00665-9

**Published:** 2021-01-21

**Authors:** Shuangyi Sun, Zhen Xie, Keting Yu, Bingqian Jiang, Siwei Zheng, Xiaoting Pan

**Affiliations:** 1 Wenzhou Medical University, Wenzhou, 325035 Zhejiang Province China; 2 The First Affiliated Hospital of Wenzhou Medical University, Wenzhou, 325035 Zhejiang Province China

**Keywords:** COVID-19, Epidemic: healthcare system, High-tech, Internet hospitals: post-epidemic era

## Abstract

With the ongoing COVID-19 outbreak, healthcare systems across the world have been pushed to the brink. The approach of traditional healthcare systems to disaster preparedness and prevention has demonstrated intrinsic problems, such as failure to detect early the spread of the virus, public hospitals being overwhelmed, a dire shortage of personal protective equipment, and exhaustion of healthcare workers. Consequently, this situation resulted in manpower and resource costs, leading to the widespread and exponential rise of infected cases at the early stage of the epidemic. To limit the spread of infection, the Chinese government adopted innovative, specialized, and advanced systems, including empowered Fangcang and Internet hospitals, as well as high technologies such as 5G, big data analysis, cloud computing, and artificial intelligence. The efficient use of these new forces helped China win its fight against the virus. As the rampant spread of the virus continues outside China, these new forces need to be integrated into the global healthcare system to combat the disease. Global healthcare system integrated with new forces is essential not only for COVID-19 but also for unknown infections in the future.

## Background

COVID-19 has spread rapidly and enveloped most countries, becoming a once-in-a-century global health crisis [1]. The number of cases diagnosed with COVID-19 has risen exponentially, with 19,718,030 confirmed cases worldwide and 728,013 deaths recorded as of August 10, 2020 [2]. Policymakers and hospitals did not have sufficient time to accommodate the sudden variation and adjust their response, resulting in unprecedented disruption to the global healthcare system. Owing to major public health risks it posed to global health, the outbreak was declared as a pandemic by the World Health Organization (WHO) on March 11, 2020 [3].

Whether the capacity of the current healthcare system can keep pace with the pandemic is a serious concern. Countries outside China, including India, Brazil, and the United States, are potentially losing control of the pandemic [2]. The United States declared a state of national emergency on March 13, 2020; Spain followed suit on March 14, 2020.

**Fig. 1:**
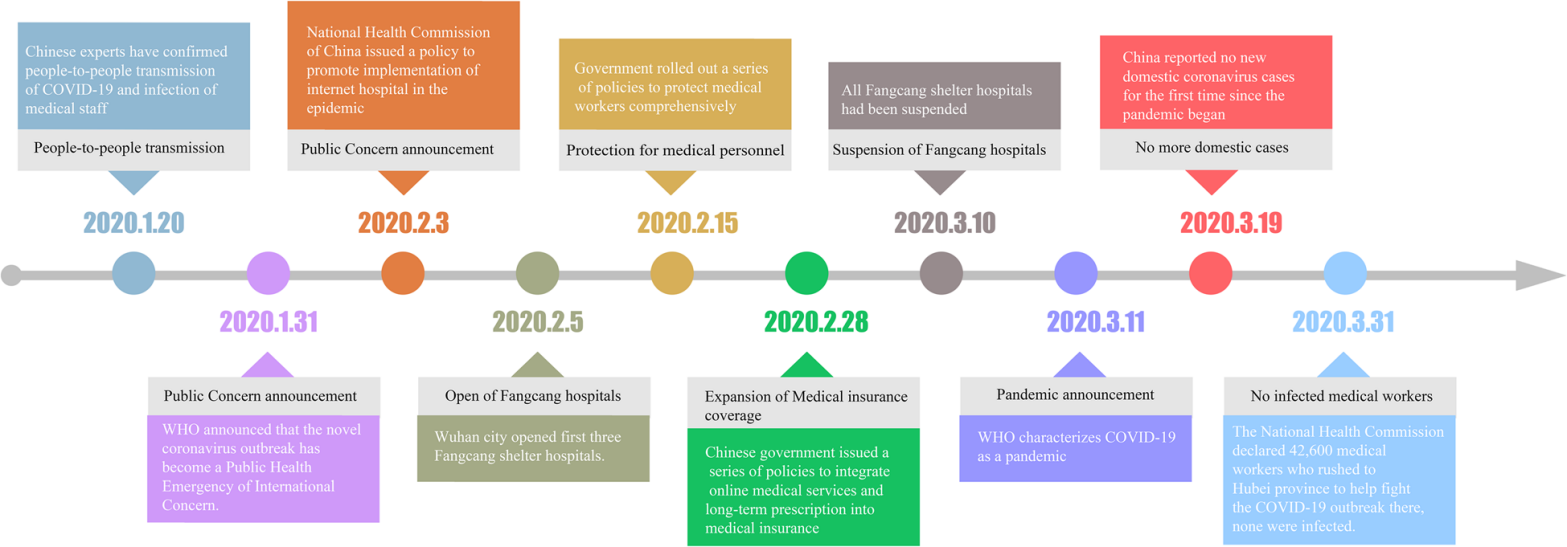
Essential events during the fight against COVID-19 in China

In most countries and regions struck by COVID-19, hospitals and other practices have reached their maximum capacity and continue to experience severe shortages of medical resources. According to the Centers for Disease Control and Prevention (CDC) website, more than 91,000 medical workers across the United States have been diagnosed with COVID-19 as of early July [4]. Moreover, almost all forefront medics worldwide are suffering from both physical and psychological exhaustion. These situations reveal that global healthcare systems are likely to operate beyond their maximum capacities for several months [5]. (Fig. 1).

## Methodology

Searches using PubMed and the WHO website were conducted to gather the number of deaths from COVID-19 and confirmed cases, reports describing disruption to the global healthcare system, and the progress of the healthcare system in the fight against COVID-19. Our search terms included “COVID-19” and “healthcare system.” Data used in this review were extracted from relevant papers.

## Findings

### China CDC failed to detect and respond to epidemic at the early stage

In early December 2019, several doctors in Wuhan discovered a kind of unexplained pneumonia [6]. The overwhelming majority of the public had limited knowledge of the novel coronavirus and low awareness of its severity and strong infectivity at the very beginning of the outbreak. The reason is that the Chinese Center for Disease Control and Prevention (China CDC) failed to detect, inspect, and respond to epidemics, which would have involved notifying the health authority without delay at the early stage [7].

**Fig. 2:**
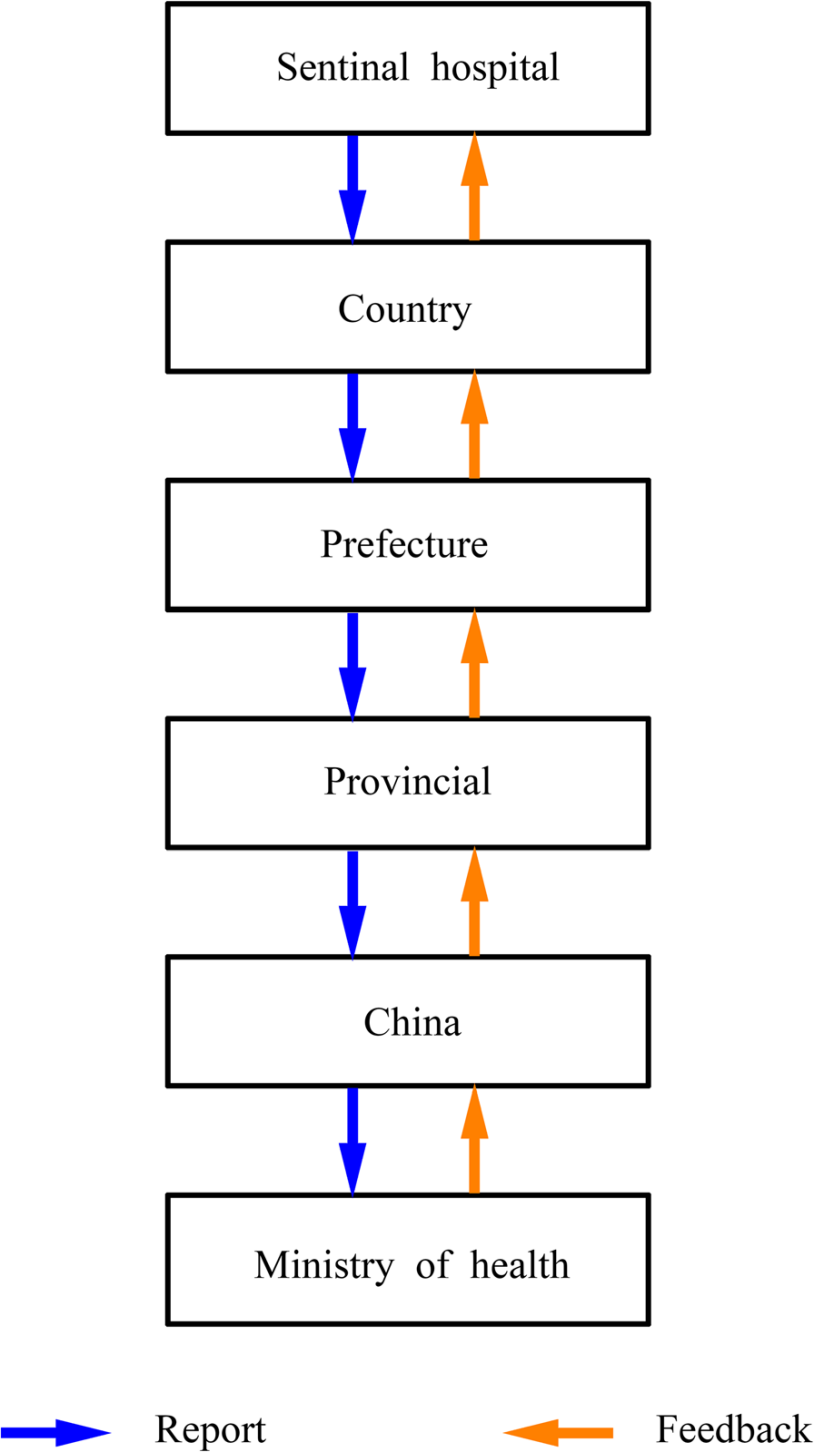
Structure of China CDC

China CDC is a three-layer system consisting of China CDC, provincial and prefecture CDC, and county CDCs [7]. If the sentinel hospital detects suspected cases, it is expected to report the disease information, level by level, to the county CDC, prefecture and provincial CDC, and China CDC, as well as the Ministry of Health (Fig. 2). However, in this process, the local epidemic data are redundantly passed on to a higher level and undergo stringent analysis. Inflexible cooperation conceivably leads to increased exacerbation of risks, impeding the prevention and control of the disease at a later stage.

In addition, with limited financial support and inadequate national administrative mandates, China CDC is currently only a technical sector, restricting its handling of a large-scale public health emergency such as the COVID-19 crisis.

### Community hospitals were overshadowed by large hospitals

The expansion of the COVID-19 epidemic in Wuhan reached the stage of community transmission in early January 2020 [8]. Community hospitals were supposed to act as gatekeepers to inhibit the large-scale transmission of the virus [9]. However, the outbreak exposed the weak capacity of community hospitals, including outdated equipment, low competency of doctors, as well as limited ability for virus testing and monitoring. Most patients refused to go to even nearby community hospitals.

The poor state of community hospitals also caused problems in the cities. Public distrust in the competence of community physicians and the quality of diagnostic facilities prompted many patients to instead visit large hospitals for diagnosis and treatment [10]. This situation resulted in cross-infection, further overwhelming hospitals with large numbers of patients. Under these conditions, China failed to contain the virus within the community, and community-wide transmission has escalated [10].

### Brick-and-mortar hospitals could not meet the demand for medical services

Physical hospitals failed to efficiently function both for patients infected with COVID-19 and those with other diseases during the epidemic.

In some of the hardest-hit cities, medical resources were not available for every COVID-19 patient. With numerous confirmed cases, thousands of patients with mild to moderate symptoms of COVID-19 had to be sent home for isolation and observation, potentially exposing their family members to the disease and prompting high rates of intra-family infection.

A considerably wider hospital-related transmission of the virus was reported in physical hospitals [11]. Patients with atypical clinical manifestations were also contagious during the incubation period [12]. Similarly, frontline healthcare workers were exposed to a high risk of infection, increasing the transmission to patients hospitalized for other diseases. To control the nosocomial spread of the virus, a large number of physical hospitals postponed or canceled outpatient appointments [6]. These drastic containment and mitigation measures significantly affected routine medical services for the public, preventing fragile patients and chronic pain patients from accessing necessary services [13]. For instance, patients with cardiocerebrovascular diseases, chronic renal failure, and diabetes mellitus, among others, encountered problems seeking maintenance treatment. This scenario is not limited to China and extends worldwide [14].

### Healthcare workers could not meet the massive influx of patients with COVID-19

Always standing at the forefront, healthcare workers represent a major force in tackling diseases and saving lives. At the end of 2019, there were only 6.41 public health professionals per 10,000 population, creating a substantial shortage of medical workers. The sudden epidemic further overburdened the Chinese healthcare workers [15–17]. The failure of healthcare workers to meet the tremendous influx of patients with COVID-19 was widely reported.

With rising cases of infection, the working environment was extremely difficult for frontline healthcare workers in China. One critical concern was the dire shortage of personal protective equipment (PPE) for healthcare workers [5]. Frontline health workers were reported to wait for PPE while already treating patients, increasing their risk of infection. Data from the National Health Commission of the People’s Republic of China revealed that more than 3000 healthcare workers had been infected as of early March, 2020, 62 of which died. Health workers becoming ill or self-isolating further limited the workforce. During the peak of the epidemic, the shortage of health workers was quite severe.

Most health workers suffered from emotional exhaustion. Moreover, emotional disturbance, fear, and anxiety over the possibility of contaminating their families were frequently reported during the epidemic. A recent study involving 1563 health workers revealed that 50.7% of the respondents reported depressive symptoms, 44.7% suffered from anxiety, and 36.1% experienced sleep disturbances [18].

### Medical resources were direly deficient

As the epidemic progressed, almost all tertiary and secondary hospitals across the country experienced a serious dearth of medical resources. Ventilators, gloves, surgical masks, disposable isolation gowns, eye protection, essential medicines, and equipment were inadequate and far from meeting the demand. The number of beds available in the hospitals designated for treating coronavirus patients was insufficient [19]. The scarce supply of qualified medical resources further aggravated the healthcare burden.

The COVID-19 outbreak coincided with the Spring Festival when most manufacturers and distributors were on holiday, exacerbating the shortage of protective medical supplies [20]. In addition to the supply shortage, some items were either substandard or expired. In China, the availability and accessibility of basic healthcare resources substantially varied among regions, and the disparity between the supply and demand of resources for disease prevention and control remained prominent, particularly in the epicenter of the outbreak [21].

Studies suggest a potential association between mortality from COVID-19 and the availability of medical resources [22]. Thus, the problem related to medical resources needs to be addressed to meet the rapid increase in the number of infected cases.

## Discussion

### Fangcang hospitals responded efficiently to the epidemic

Owing to the lack of hospital beds for patients infected with COVID-19, some public places, such as conference centers and stadiums, were converted into shelters also known as “Fangcang hospitals.” [23]

Fangcang hospitals have several advantages and thus are crucial in the control of the epidemic [19]. First, they are characterized by rapid construction, which facilitates the immediate admission and treatment of patients. Moreover, they only need redesign and medical devices [24]. Second, converted from venues, Fangcang hospitals allow the large-scale provision of beds to admit patients and thereby relieve the burden on the healthcare system. Last, they exhibit improved utilization of medical resources. Fangcang hospitals only accommodate patients with no severe symptoms, requiring fewer physicians and nurses [25]. Moreover, admission and diagnosis are unified, simplifying the entire process [26]. Consequently, the utilization and distribution of medical resources are optimized.

Fangcang hospitals functioned efficiently during the COVID outbreak. First, they isolated patients with mild to moderate symptoms [27], allowing the treatment of every patient and the prevention of possible transmission. Patients with mild to moderate symptoms who are quarantined at home are likely to expose their family and relatives to risk [26, 28]. Second, Fangcang hospitals implemented a system involving a simple pathway of referral and transfer. The temperature, respiration rate, blood pressure, and oxygen saturation of the patients were measured multiple times daily. Patients whose health status worsened were immediately transferred to higher-level hospitals [27]. Last,Fangcang hospitals integrated social engagement and physical support to patients in their treatment program. These mechanisms served as communities for patients, where they could participate in social activities, such as reading, dancing, watching TV, and celebrating birthdays [29, 30]. In addition, the patients were provided physical comfort by the health workers.

Measures such as the construction of Fangcang hospitals and quarantine, among others, led to a large reduction in the increase in the number of patients. More than 12,000 patients were cured during their operation. As of March 10, all Fangcang hospitals in Wuhan had been closed [31].

The number of confirmed cases is still rapidly rising worldwide, posing a threat to health care systems in countries other than China. Specifically, in Italy, traditional hospitals were overwhelmed with ill patients, causing shortages of hospital beds [32].

China has helped other countries such as Italy, Serbia, and Iran construct and operate Fangcang shelter hospitals in their fight against COVID-19 [33–35].

Similar to Fangcang hospitals, field hospitals have been used in the United Kingdom and Spain, and makeshift hospitals have been constructed in Iran to help attending to the isolated patients [36–39].

### Improved attitudes toward healthcare professionals

In their fight against the epidemic, the overwhelming majority of doctors carry the responsibility of safeguarding the health of all people in China. Medical personnel have received increased attention and recognition through this outbreak, including the acknowledgment and protection by the State and the support and respect of the people. With such recognition, most medics have devoted themselves to medical and health care services. A total of 42,000 health workers across the country have assisted Hubei province regardless of the high risk of cross-infection [40].

During this epidemic, further measures were promulgated to provide incentives to healthcare workers and protect them in all aspects, including subsidies and allowances, work-related injury compensation, psychological health services, and daily needs, among others [41, 42]. Beyond legal right protection, these measures reflect the appreciation of the country for their contributions.

Apart from policy protection measures, a cultural shift in the social status of doctors and attitude toward doctors has been observed. Both doctors and patients have been understanding and supportive of one another in their fight against the virus. Doctors and nurses have received national recognition for their pivotal role in halting the spread of the disease and have gained public support and respect. Cities across the country have lit up their landmark buildings for medical workers, displaying their faces and stories, applauding them as “the most admirable people in the new era.”

### Internet hospitals helped eased the burden on offline clinics

To solve the dilemma between the demand for medical care and the inaccessibility of medical services, the Chinese government has issued a series of policies, such as the incorporation of online medical services and long-term prescription into medical insurance, to empower Internet hospitals and thereby address public health emergencies [11, 43]. With these favorable policies, Internet hospitals such as WeDoctor and Alibaba Health have emerged, gaining considerable public interest [11].

An Internet hospital is a platform for the delivery of approved remote medical services via Internet technologies for consultation, treatment and diagnosis, as well as prescriptions [44]. An Internet hospital widely varies from telemedicine in Western society [45]. Most Internet hospitals are based on physical hospitals where patients receive almost the same medical services as those in physical hospitals, such as prescriptions and health insurance programs [11]. The COVID-19 epidemic is the first instance in which Internet hospitals were involved in a public health emergency caused by an infectious disease. At the beginning of the epidemic, 42.3% of physical hospitals nationwide established their Internet-based hospitals, alleviating the flood of people in physical hospitals [46].

Internet hospitals have three advantages that render them suitable to support the fight against the infectious disease. (i) First, the platform operates via non-contact treatment. Internet hospitals provide remote online medical services for patients at home or anywhere, minimizing face-to-face contact with susceptible populations and lowering the occurrence of nosocomial cross-infection. Second, Internet hospitals provide an optimized resource distribution. Internet technologies help rebalance the distribution of medical services, linking better medical resources in East China with demands in the central and western areas [44, 47]. The Internet enables people to overcome geographical obstacles to health care—that is, rural areas are given the same access to health care as that of urban areas [44]. Patients only need to visit their community health center and village clinic or a pharmacy near their area to consult with skilled doctors based in big cities and obtain a diagnosis [48]. Third, they perform with high efficiency at a low cost. Most Internet hospitals provide online services continuously and have an extensive reach. Using this platform, patients and doctors only need a computer, a laptop, or a smart phone.

Internet hospitals play an indispensable role in four major aspects. First, they offer different types of online consultations, including epidemic-related counseling, psychological counseling, and home quarantine guidance. These online consultations considerably alleviate social panic [14]. Data from WeDoctor and haodf.com indicate that 20% of their online medical consultations consist of COVID-19 and essential epidemic-protective skills, as well as guidance for home quarantine [49]. Furthermore, doctors and patients, as well as the public seek online support to address their mental health needs [26]. Second, through Internet hospitals, patients with chronic or common diseases can complete their regular follow-up consultations without delay. Even under severe conditions, online doctors can instruct patients to visit offline clinics as safely as possible. Furthermore, real-time telemedicine from multidisciplinary experts enhances the efficiency of treatment for acutely ill patients in Wuhan City, sharply decreasing the mortality rate. Last, the administration of medication, prescription, as well as contactless delivery extend the use of Internet hospital.

While COVID-19 has spiraled into a global health crisis, several Internet hospitals in China, such as WeDoctor and AliHealth, have extended their international online services to assist some of the hardest-hit or resource-limited countries (Table 1).

**Table 1:**
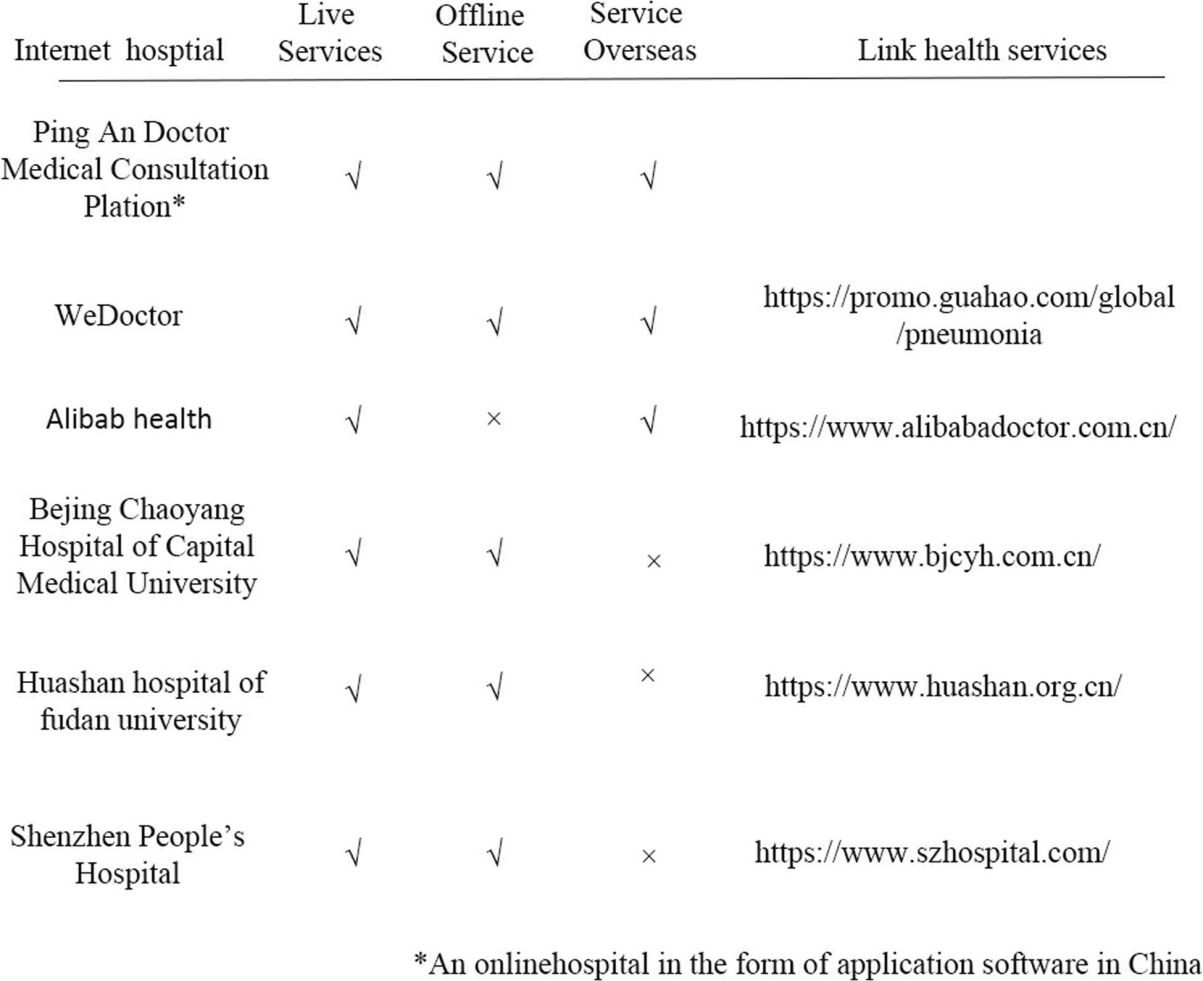
Widely used Internet hospitals and their services in China

COVID-19 has, within a short period, accelerated the growth of Internet hospitals as a public health and social distancing measure. In the post-epidemic era, the standard and quality of Internet hospitals have to be improved to promote routine medical care in non-crisis situations.

### High-tech lends helping hand in the outbreak fight

China has flexibly and swiftly used technology and innovation to respond to the novel coronavirus crisis. Several Internet-based companies, such as Alibaba Group, Baidu Inc., and Tencent, have joined the battle at the outset. They have leveraged advanced technologies, including 5G, big data analysis, cloud computing, and artificial intelligence (AI) to empower health care [50].

AI and 5G have been extensively applied in a large number of hospitals, assisting frontline medical workers [50]. Moreover, 5G-enabled robots have been installed at hospitals to offer various automated medical services, including drug delivery, measurement of mobile patrol temperature, hospital disinfection and cleaning, route guidance, and other repetitive tasks [51]. These smart robots, which effectively reduce cross-infection, are widely welcome in isolated wards. AI-coupled point-of-care (POC) diagnosis screens suspected infections and closely monitor changes in the physical condition of patients to contain the spread of the disease in hospitals [52]. The AI-enabled auxiliary diagnostic system enhances diagnostic accuracy and speed while protecting medical workers. Alibaba Group, the Chinese e-commerce giant, has developed an AI system that can detect the presence of coronavirus with 96% accuracy in 20 s by assessing computed tomography scans, in contrast to the 15-min duration required to evaluate humans [53]. The health code is also a disease prevention technology based on big data. Downloaded on mobile phones, the health code shows the places visited by the phone owners and the risk of their close contact with COVID-19 patients, thus lowering the risk of infection for their neighbors.

### Healthcare system in the post-epidemic period

Early detection and reporting are key to curbing the spread of an epidemic. Relying solely on large public hospitals is not an efficient means of preventing the disease. The COVID-19 outbreak has exposed some of the weaknesses of community hospitals, which are supposed to serve as gatekeepers for the health of their residents. These weaknesses include (1) the relatively limited capacity of their primary care services, which prevents patients from visiting even nearby community hospitals for diagnosis and treatment and (2) the lack of fever clinics or qualified personnel, or even beds in many community-level hospitals and clinics in China [54, 55].

Given the aforementioned scenario, consistent efforts are needed to empower community hospitals and address public mistrust. Feasible approaches are as follows: (1) Setting up standard fever clinics and fever screening checkpoints to improve early warnings of infectious diseases; (2) Providing medical staff with regular training to enhance their ability to detect infectious diseases during regular medical services; (3) Upgrading infrastructure and equipment, strengthening reserves of medical equipment, and boosting primary care services; (4) Improving the deployment of Internet and Information Technology, thus narrowing the gap between community hospitals and well-equipped large public hospitals; (5) Substantial reform of the general practitioner system (healthcare workers with adequate knowledge in all branches of medicine) and the establishment of an effective primary diagnostic process and a two-way referral system.

In response to the prevailing public distrust of medical personnel, feasible approaches are suggested: (1) The antagonistic pattern of economic interests between doctors and patients should be broken, and a social medical security system and a medical resource distribution system that is fairn and just should be established; (2) The monopoly of medical information should be reduced, and a mandatory medical information disclosure system should be established; (3) Doctor-patient communication should be given importance, and the information gap between doctors and patients should be narrowed; (4) The construction of medical ethics should be strengthened, and the professional image of the medical staff should be maintained.

Simultaneously, while Internet hospitals have proven to be indispensable in responding to the epidemic, their healthcare benefits beyond the situation have also become evident.

However, some of these benefits are coupled with barriers and challenges that must be addressed. These problems may be attributed to several factors, such as medical reimbursement, willingness of clinicians, and the staff of the Internet hospital.

Medical insurance is an integral part of healthcare; thus, online medical reimbursements have to be popularized. With the integration of medical insurance into Internet-based healthcare, a closed loop is formed. Patients can enjoy online consultations, online reimbursement, and drug deliveries without leaving their homes. Patients in rural areas via can be connected to improved medical resources via Internet hospitals.

Nonetheless, not all healthcare workers have a thorough knowledge of online healthcare. At a time of need, a large number of clinicians revert to the their previous means of interacting with the traditional healthcare system. Online healthcare need to be integrated into medical education, driving their significant inclusion in clinical practices in the future.

Last, to further promote the use of online healthcare in the post-epidemic era, Internet hospitals have to set a better mode and standard. Country regulations, quality of healthcare services, stable social networks, patient privacy, and data security remain top priorities. Thus, Internet hospitals can bring more social value and influence into the entire healthcare system.

Overall, human victory over epidemics depends on technological innovation and scientific development. Indeed, emerging technologies such as 5G, AI, and Internet hospitals played a key role in containing the disease. However, these new forces were not included in the emergency action plan for epidemics. Thus, they should be adapted to the new normal coping mechanism for possible epidemics in the future.

## Conclusions

This study presents a comprehensive summary of the approach taken by the government to contain COVID-19 in China. It also cites the weaknesses of the healthcare system struggling to curb an emerging epidemic (namely, failure to detect early the spread of the disease and medical resource shortage) and the methods by which these weaknesses have been addressed and can be addressed in the future, such as adapting 5G, AI, and Internet hospitals to the new normal coping mechanism for possible epidemics.

## Data Availability

All materials cited in this publication and consulted research can be consulted in the cited references. We did not consult any data bases that are privately owned or inaccessible to the public.
